# Impact of frailty on the management of patients with gynecological cancer aged 80 years and older

**DOI:** 10.1007/s00404-020-05807-9

**Published:** 2020-10-03

**Authors:** Elisabeth Reiser, Nina Pötsch, Veronika Seebacher, Alexander Reinthaller, Friedrich Wimazal, Edith Fleischmann, Michael Krainer, Reinhart Horvat, Stephan Polterauer, Christoph Grimm

**Affiliations:** 1 Division of General Gynecology and Gynecologic Oncology Department of Obstetrics and Gynecology, Comprehensive Cancer Center, Medical University of Vienna, Vienna, Austria; 2 Karl Landsteiner Institute for General Gynecology and Experimental Gynecologic Oncology, Vienna, Austria; 3 Clinic for General Anesthesiology, Intensive Care and Pain Management, Medical University of Vienna, Vienna, Austria; 4 Clinical Division of Oncology, Department of Internal Medicine I, Medical University of Vienna, Vienna, Austria; 5 Department of Pathology, Medical University of Vienna, Vienna, Austria

**Keywords:** Frailty, Gynecological malignancy, Gynecological cancer, Survival, Complication rate

## Abstract

**Purpose:**

To assess the impact of frailty on compliance of standard therapy, complication, rate and survival in patients with gynecological malignancy aged 80 years and older.

**Methods:**

In total, 83 women with gynecological malignancy (vulva, endometrial, ovarian or cervical cancer) who underwent primary treatment between 2007 and 2017 were retrospectively analyzed. Frailty index was calculated and its association with compliance of standard treatment, peri- and postoperative mortality and morbidity, and survival was evaluated.

**Results:**

Frailty was observed in 24.1% of cases. Both frail and non-frail patients were able to receive standard therapy in most cases − 75.0% and 85.7%, respectively (*p* = 0.27). Frail patients did not show an increased postoperative complication rate. Frail patients had shorter 3 years overall survival rates (28%) when compared to non-frail patients (55%) (*p* = 0.02). In multivariable analysis high frailty index (Hazard Ratio [HR] 12.15 [1.39–106.05], *p* = 0.02) and advanced tumor stage (HR 1.33 [1.00–1.76], *p* = 0.05) were associated with poor overall survival, but not age, histologic grading, performance status, and compliance of standard therapy.

**Conclusion:**

Majority of patients was able to receive standard therapy, as suggested by the tumor board, irrespective of age and frailty. Nonetheless, frailty is a common finding in patients with gynecological malignancy aged 80 years and older. Frail patients show shorter progression-free, and overall survival within this cohort.

**Electronic supplementary material:**

The online version of this article (10.1007/s00404-020-05807-9) contains supplementary material, which is available to authorized users.

## Introduction

Life expectancy is consistently rising in developed countries. The rate of women older than 80 years is increasing [1]. Chronologic age can differ from biologic age [2]. Frailty is a chronically underdiagnosed condition reaching a prevalence ranging from 11 to 43% in the general population [3, 4]. In women in general and in female cancer patients in particular the prevalence of frailty is even higher [5, 6]. Frailty is a geriatric syndrome characterized by reduced homeostatic reserve, reduced capacity of coping with acute and environmental stressors, and poses the individual to higher risk of negative health-related outcomes [7]. Frail patients show increased risk of falls, disability, hospitalization and death [8–10]. Cancer treatments including surgery, chemotherapy, and radiotherapy are possible stressors that can cause the transition to an unstable condition of frail patients.

To individualize the treatment of the oldest old, exact assessment of physical status and ability to tolerate treatment are needed. It seems that old patients without frailty are able to tolerate radical treatment without increased risk of complications [2]. Frailty can be measured and objectively assessed by several tools such as the frailty index, the comprehensive geriatric assessment, and the Fried scale [8, 11–13]. These tools are able to predict surgical complications, length of hospital stay, hospital readmission, and survival [8, 9, 14]. Frailty is a more accurate predictor of these events than chronologic age. In a recent study frailty was independently associated with worse surgical outcome and higher postoperative complication rate in patients with epithelial ovarian cancer. Frailty was associated with a decreased likelihood for the initiation of chemotherapy within 42 days after surgery and subsequently also associated with poorer overall survival [2].

So far, no study reported the influence of frailty in women aged 80 years and older with gynecological malignancy. The aim of this study was to ascertain the prevalence of frailty by using the frailty index in a group of women with gynecological malignancy aged 80 years and older undergoing primary treatment and to report the association between frailty and compliance of standard treatment, surgical morbidity, and survival.

## Materials

### Patients

In total, 83 consecutive patients undergoing primary treatment for vulva, endometrial, ovarian or cervical cancer aged 80 years and older were included in the present retrospective analysis. The institutional review board of the Medical University of Vienna approved the present study (institutional review board number 2217/2017). All patients were treated at the Department of Obstetrics and Gynecology, Division of General Gynecology and Gynecologic Oncology, Medical University of Vienna, Austria, between January 2007 and December 2017. All patients, who are treated at this department, sign an informed consent to use their data for retrospective studies.

Information about patients’ demographics and comorbidities as well as data about intraoperative and postoperative complications was obtained using electronic chart review. Postoperative complications were recorded for the first 30 days after surgery and graded according to the Clavien–Dindo Classification [15]. Information about adjuvant treatment included the date of chemotherapy/irradiation initiation, possible dose reductions, date and location of progression, vital status, and date of last follow-up or death were also recorded. The 2009 International Federation of Gynecology and Obstetrics (FIGO) classification system was used [16, 17]. Patients were treated according to international guidelines as reported previously [18]. If patients received the therapy suggested by the institutional tumor board and accomplished this therapy, it was considered as ‘compliance of standard therapy’.

### Frailty index

To calculate the frailty index deficit variables were abstracted from the medical record. Activities of daily living, exercise tolerance, and need for assistance were documented during routine assessment. Prior to treatment initiation, all patients were seen by an internal specialist and an anesthesiologist. Additional information for frailty index assessment including comorbidities and bodymass index was obtained from electronic chart review. Frailty index was based on 31 items that have been used to categorize frailty in previously published studies [13, 19] [Supplemental Table S1]. One item—malignancy—needed to be removed from the list of comorbidities as this applies to all patients in our cohort. The practice of removing items from the index that are applicable for the whole cohort has been performed before [2]. Frailty index was calculated only if less than two items were missing. In general, the individual reached score was divided by the total number of non-missing items. For example, if a patient had information on 29 of 31 items and 5 deficits/comorbidities that each scored 1 point, then the frailty index would be 5/29 (0.17). An already established cut-off was used to classify patients’ frailty: patients with a frailty index ≥ 0.25 were considered as frail [4].

### Statistics

Values are given as median (interquartile range [IQR]) and mean (standard deviation [SD]). Parameters were compared between frail and non-frail patients using *t*-tests, chi-squared or one-way ANOVA-tests where appropriate. Survival probabilities were calculated by the product limit method of Kaplan and Meier. Differences between groups were tested using the log-rank test. The results were analyzed for the endpoint of overall survival. Survival times of patients that were still alive at the last follow up visit were censored with the last follow-up date. Univariate survival analysis was performed using log-rank test and Cox Regression analysis. Kaplan–Meier survival curves were computed. Multivariable analysis was conducted using Cox regression including as independent variables frailty index, tumor stage (FIGO IV vs. FIGO III vs. FIGO II vs. FIGO I), and ECOG (4 vs. 3 vs. 2 vs. 1 vs. 0) and cancer type (vulva vs. endometrial *vs.* ovarian vs. cervical). *p* values < 0.05 were considered statistically significant. For all the statistical analyses SPSS statistical software system version 25.0 was used.

## Results

In 83 patients of 95 identified patients with gynecological malignancy frailty index could be calculated (37 with vulvar cancer, 35 with endometrial cancer, 8 with ovarian cancer and 3 with cervical cancer). Missing information especially regarding activities of daily living caused missing values in 12 patients. Tables 1, 2 show patients’ characteristics and treatment outcomes. Interestingly only 36.1% of all patients had FIGO stage I disease and the majority of patients had disease beyond the primary origin. Frailty was seen in 24.1% of cases.

**Table 1:** Patients’ characteristics (*N* = 83)

Parameter	*N* (%) or mean (SD)
Frailty index	0.19 (0.16)
Non-frail, FI < 0.25	63 (75.9)
Frail, FI ≥ 0.25	20 (24.1)
Age, years	84.2 (3.5)
ECOG performance status	
0	48 (57.8)
1	24 (28.9)
2	6 (7.2)
3	4 (4.8)
4	1 (1.2)
BMI (kg/m^2^)	24 (4.3)
Cancer type	
Vulva cancer	37 (44.6)
Endometrial cancer	35 (42.2)
Ovarian cancer	8 (9.6)
Cervical cancer	3 (3.6)
FIGO stage	
I	30 (36.2)
II	25 (30.1)
III	19 (22.9)
IV	9 (10.8)
Number of comorbidities	3.3 (1.7)
Number of long-term medications	5.2 (3.4)

*SD *standard deviation, *FI *frailty index, *ECOG *Eastern Cooperative Oncology Group, *BMI *Body Mass Index, Performance status, *FIGO *International Federation of Gynecology and Obstetrics

**Table 2:** Information on treatment of patients with gynecological cancer aged 80 and older (*N* = 83)

Parameter	*N* (%) or mean (SD)
Standard therapy	
Yes	69 (83.1)
No	14 (16.9)
Type of primary treatment	
Surgery	68 (81.9)
(Chemo)radiation	15 (18.1)
Complete resection	
Yes	58 (85.3)
No	10 (14.7)
Duration of hospital stay after surgery, days	11.2 (6.9)
Mortality after surgery within 30 days	None
Type of adjuvant treatment	
Chemotherapy	9
Radiation	18
None	56
Follow-up time, months	29.2 (27.2)
Status	
Alive	38 (45.8)
Dead	45 (54.2)
Recurrence	
Yes	24 (28.9)
No	59 (71.1)
Cause of death	
Cancer related	17 (37.8)
Other cause	23 (51.1)
Unknown	5 (11.1)

*SD *standard deviation

Patients were characterized into non-frail (frailty index < 0.25) and frail (frailty index ≥ 0.25). Patients’ characteristics broken down by frailty are presented in detail in Table 3. There was no difference in compliance of standard therapy, postoperative complication rate, and duration of hospitalization between frail and non-frail patients (Table 3). Out of nine patients with planned adjuvant chemotherapy, six patients received the planned dosage. There was no difference between frail and non-frail patients in completion of the planned dosage (*p* = 0.70).

**Table 3:** Characteristics of frail and non-frail patients during the postoperative interval

Parameter	Non-frail FI < 0.25 *N* (%) or mean (SD)	Frail FI ≥ 0.25 *N* (%) or mean (SD)	*p *value
Standard therapy			0.27^a^
Yes	54 (85.7)	15 (75.0)	
No	9 (14.3)	5 (25.0)	
Complete resection rate			0.49^a^
Yes	47 (83.9)	11 (91.7)	
No	9 (16.1)	1 (8.3)	
Postoperative complications			0.40^a^
No complications	40 (71.4)	10 (83.3)	
Any complications	16 (28.6)	2 (16.7)	
Type of complication			
Minor complications^c^	10 (66.7)	0 (0)	0.07^a^
Major complication^d^	5 (33.3)	2 (100)	
Duration of hospital stay, days	10.9 (6.7)	12.3 (7.8)	0.52^b^
Time to start with adjuvant treatment, days	40.2 (25.1)	35.0 (9.9)	0.78^b^

*SD *standard deviation

^a^*χ*^2^ test

^b^*t*-test

^c^Clavien Dindo Classification ≤ 2

^d^Clavien Dindo Classification > 2

**Fig. 1:**
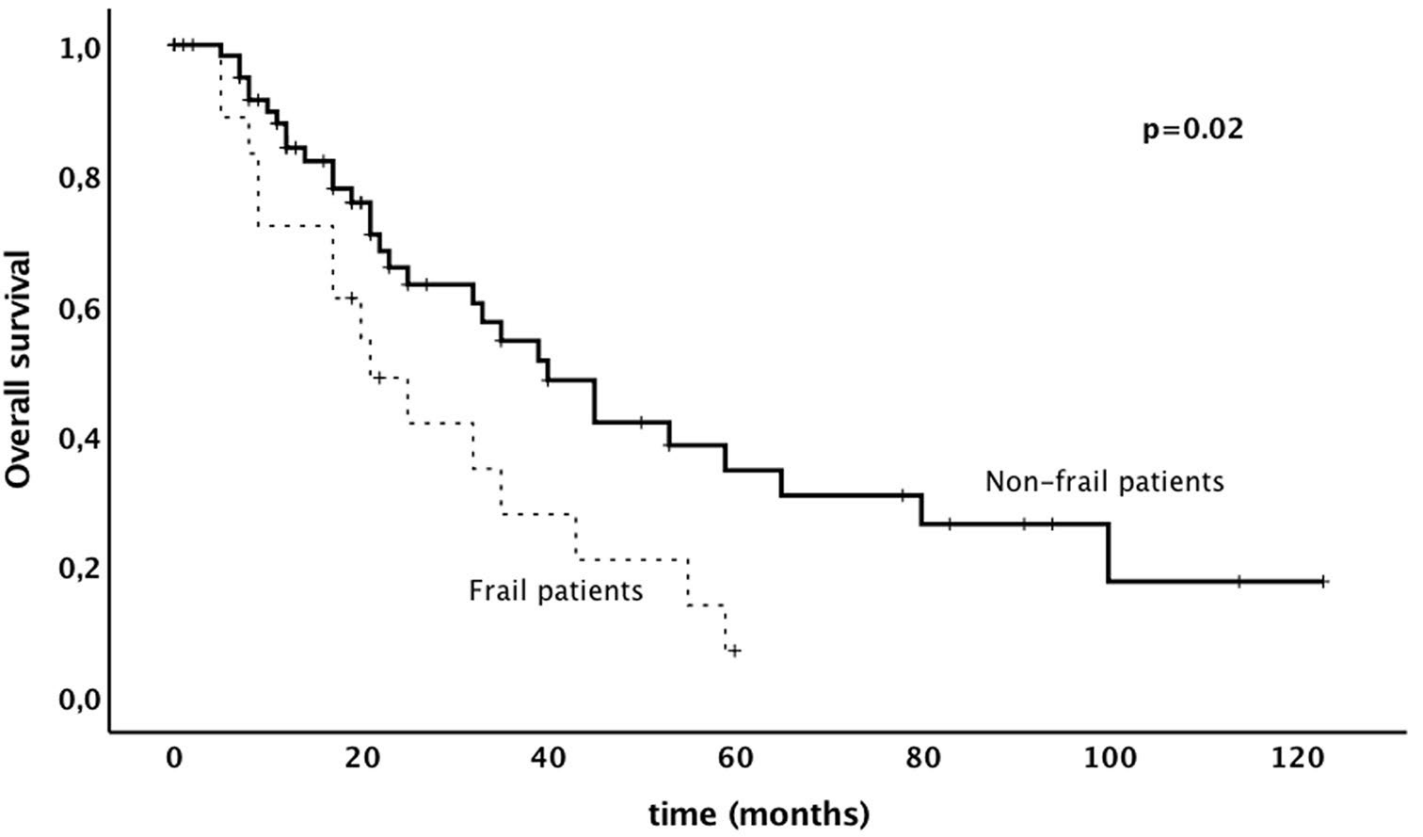
Progression-free survival in patients aged 80 and older with gynecological malignancy depending on frailty

**Fig. 2:**
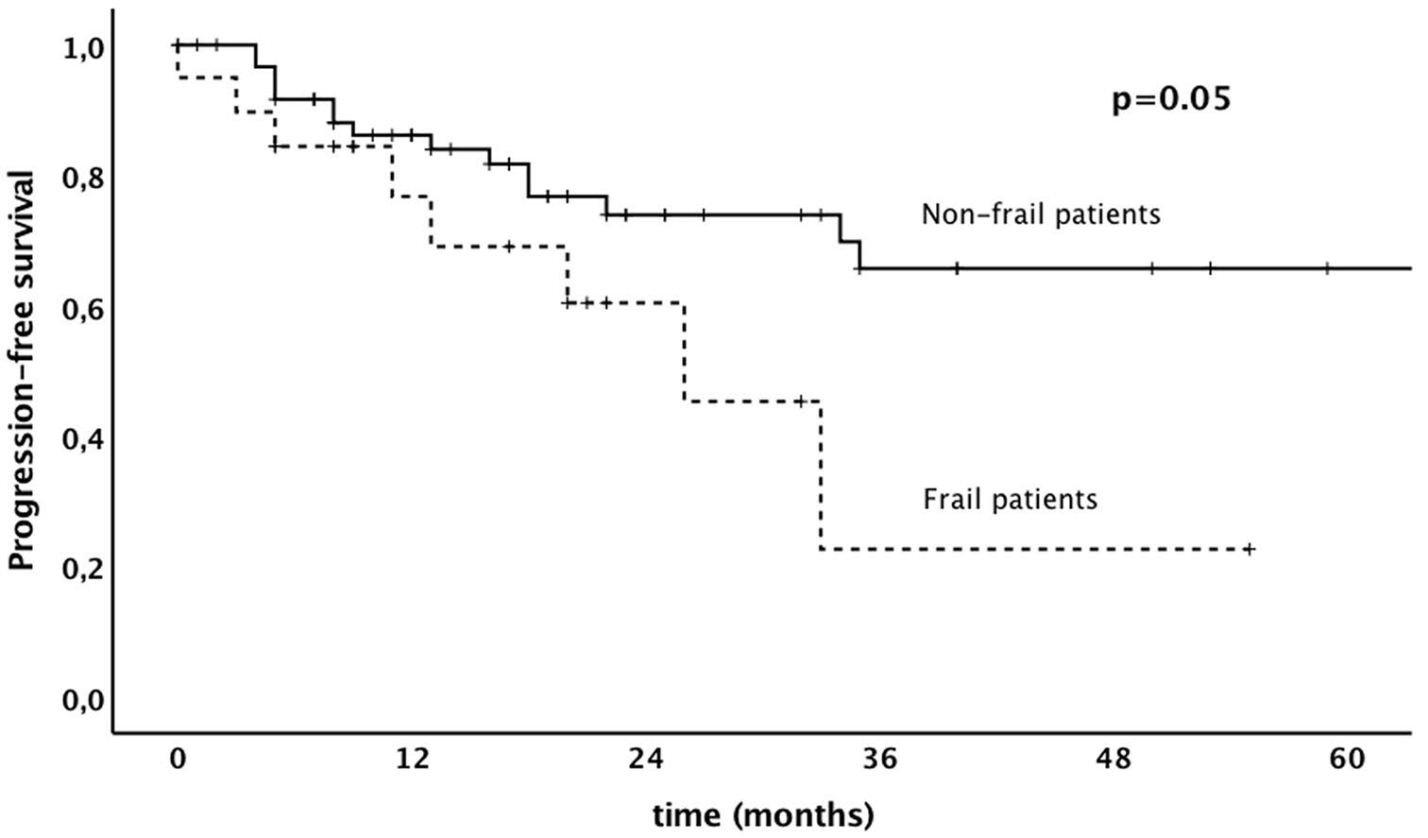
Overall survival in patients aged 80 and older with gynecological malignancy depending on frailty

Results of uni- and multivariable analysis are shown in Table 4. In univariate analysis higher frailty index was associated with shorter progression-free survival. In multivariate analysis frailty index and other prognostic markers were not associated with poorer progression-free survival. With respect to overall survival, frailty index was identified as an independent prognostic marker in both univariate and multivariable survival analysis [*p* = 0.05; *p* = 0.02, HR 12.15 (Confidence Interval [CI] 1.39–106.05)]. Frail patients had a significantly shorter overall survival compared to non-frail patients (3 years survival rate, 28% versus 55%, *p* = 0.02). Kaplan–Meier survival curves, showing the association between frailty and progression-free survival and overall survival, are shown in Figs. 1, 2.

**Table 4:** Uni- and multivariate analysis of prognostic factors on progression-free and overall survival of patients aged 80 and older

Parameter	Progression-free survival				Overall survival			
	Univariate^a^		Multivariate^b^		Univariate^a^		Multivariate^b^	
	*p *value	HR (95% CI)	*p *value	HR (95% CI)	*p *value	HR (95% CI)	*p* value	HR (95% CI)
Frailty index (continuous)	0.02^b^	–	0.10	9.81 (0.66–146.75)	0.05^b^	–	0.02	12.15 (1.39–106.05)
Age (continuous)	0.73^b^	–	0.90	0.99 (0.87–1.13)	0.07^b^	–	0.08	1.09 (0.99–1.19)
Cancer type (vulvar vs. endometrial vs. cervical vs. ovarian)	0.25		0.32	0.82 (0.55–1.22)	0.70	–	0.31	0.86 (0.63–1.16)
Tumor stage (FIGO IV vs. III vs. II vs. I)	0.07		0.86	1.41 (0.95–2.10)	0.10	–	0.05	1.33 (1.00–1.76)
Performance status (4 vs. 3 vs. 2 vs. 1 vs. 0)	< 0.001	–	0.77	1.09 (0.62–1.91)	0.59	–	0.60	0.89 (0.56–1.40)
Compliance of standard therapy (yes vs. no)	0.94	–	0.83	1.16 (0.29–4.63)	0.27	–	0.58	0.76 (0.29–1.98)

^a^Kaplan Meier

^b^Cox Regression

## Discussion

In the present study, majority of patients—frail and non-frail patients—was able to receive standard therapy, as suggested by the tumor board. Postoperative complications were typically mild without any death within 30 days after surgery and similar when non-frail patients were compared to frail patients. Higher frailty index was associated with poorer progression-free, and overall survival. This is the first study to investigate the prognostic value of frailty in patients aged 80 years and older with gynecological malignancy.

The life-expectancy and the percentage of women living longer than 80 years has been steadily rising within the last decades [1]. Results of previous studies already indicated that frailty reflected a valid prognostic marker for the postoperative morbidity and mortality rate as well as survival in different solid tumors, including gynecologic malignancies [2, 20, 21]. In our cohort, frail patients were able to receive standard treatment including radical surgery without recording any case of mortality within 30 days after surgery. These findings are remarkable regarding the high percentage of advanced disease and a relatively high number of comorbidities observed. This is particularly interesting as van der Ring et al. [22] showed that physicians tend to be rather reluctant in this cohort of patients with the recommendation for chemotherapy and tend to withhold chemotherapy in older patients. In clinical practice, physicians seem to rely very much on patient’s chronologiical age and comorbidities as crucial factors in decision-making for cancer patient’s treatment plan. Only 47% of medical oncologists recommended to give standard treatment to cancer patients aged 73 or older [22]. In our study, surgery was mostly offered to non-frail patients (88% versus 48%) and therefore underlying this statement. Moreover, intentional full-dose chemotherapy is rarer in patients aged 83 years compared to patients aged 65 years [23]. In our study only nine patients received adjuvant chemotherapy. Thereafter, results on dose reduction need to be interpreted with caution. Possible dose reduction in radiotherapy was difficult to abstract of patients’ charts due to imperfect documentation.

The benefit of the frailty index is its objective and reproducible assessment. Comorbidities as well as functional status can be measured quickly via simple questionnaires and, therefore, be easily incorporated into clinical practice. Although frailty is an age-related process, there is a certain proportion of non-frail patients aged 80 years and older that have the physical constitution and outcome comparable to their younger counterparts and vice versa. An objective assessment of physical status may, therefore,, represent a more precise tool to tailor treatment and not to withhold standard therapy.

In our study, even frail patients were able to receive standard therapy (75.0%). This was comparable to non-frail patients (85.7%). Frail patients were also able to receive adjuvant treatment with an acceptably low complication rate. This finding is interesting from a clinical point of view. It seems that even frail patients aged 80 years and older can be candidates for radical treatment. Presence of frailty was not necessarily associated with limited treatment. Of note, this is in contrast to a study by McCarthy et al. [20], where frailty index was successfully used for chemotherapy decision-making in patients aged 65 and older with solid tumors. Frail patients were less likely to receive standard chemotherapy and suffered more frequently from chemotherapy induced complications. In our study, the fact that patients received the therapy suggested by the institutional tumor board and accomplished this therapy was considered “compliance of standard therapy”. Due to the retrospective study design, we considered this method as precise as possible. This might explain these conflicting results and larger studies will need to be performed to elaborate.

With respect to the postoperative period, frail patients did not have a prolonged postoperative hospital stay. Moreover, we did not observe a significantly higher overall complication rate in frail patients compared to non-frail patients. However, there was a trend towards an increased number of severe complications in frail patients compared to non-frail patients. Of note, these results have to be analyzed very cautiously due to the limited number of patients and particularly small numbers in this analysis due to the rare event of major complications. Other studies found that frail patients were more likely to experience severe postoperative complications and death within 90 days of surgery [2]. Next to the limited number of patients, these controversial findings might be caused by different study inclusion criteria within the cohorts. While Kumar et al. only included ovarian cancer patients who were treated with a complex surgery and combination chemotherapy, our study was more heterogeneous including patients with various gynecological malignancies who received different treatment modalities.

Frailty was associated with shorter progression-free survival and overall survival. These results are in line with previously published data [2, 24]. In a study by Kumar et al. [2], overall survival was significantly shorter in frail patients compared to non-frail patients (median 26.5 vs 44.9). It is biologically plausible that frail patients aged 80 and older have poorer overall survival, because frailty increases the risk for adverse outcomes including falls, delirium, and disability. This is supported by the fact that most patients within our cohort died due to non-cancer related death. In other studies, the shorter progression-free survival might be caused by a less aggressive treatment administered to frail patients [2]. In the present study, standard treatment was administered in a high percentage. Reduced progression-free survival in frail patients might be caused by the higher vulnerability and decreased homeostatic reserve which results in a dramatic and disproportionate change in health state. Kumar et al. found that frail patients were likely to have low skeletal muscle quality. Other theories put forward to account for the decreased survival in older women include more aggressive cancer with advanced age, individual patient factors such as multiple concurrent medical problems, polypharmacy, poor nutrition, and limited social support [25].

An uprising question is how frailty could be reduced during the short preoperative or pretreatment period, and if this would possibly improve the outcome of frail patients. Possible targets would be resistance exercise, myostatin inhibitors, and nutrition supplementation. Especially resistance exercise seems to translate into important functional gain [26]. Moreover, frail patients might need more intense supportive therapy and might benefit from tailored supportive care after hospital discharge.

Strengths of this study are its single center study design including unselected, consecutive cases of patients aged 80 years and older with gynecological malignancy. Patients were treated in one tertiary care center. Potential limitations might include the inhomogeneity of cancer types, shortcomings in frailty index assessment due to its retrospective analysis and shortcomings typically caused by retrospective study design. Thus, the present study can only describe rare events such as major postoperative complications and prognosis but cannot provide thorough statistical analysis. Moreover, the present study cannot evaluate the predictive and prognostic impact of interventions to reduce frailty.

In conclusion, we present the first data on patients’ frailty in women aged 80 and older with gynecological malignancy, which is a common event in this cohort. The vast majority of frail patients were able to receive standard therapy with a relatively low complication rate. Nonetheless, frail patients aged 80 years and older had significantly shorter progression-free, and overall survival.

## Electronic supplementary material

Below is the link to the electronic supplementary material.

Supplementary file1 (DOCX 13 kb)
