# Supplementary material for: Impact of frailty on the management of patients with gynecological cancer aged 80 years and older
Source: Arch Gynecol Obstet. 2020 Oct 3;303(2):557–63. doi: 10.1007/s00404-020-05807-9 (PMC7858206; doi:10.1007/s00404-020-05807-9)
Supplement: Supplementary file 1 — Supplementary file1 (DOCX 13 kb) [file 404_2020_5807_MOESM1_ESM.docx]

**Supplemental Tables**

**S1.** Frailty Deficit Index

| Deficit | Assigned points |
| --- | --- |
| 1. Need help preparing meals | No=0, Yes=1 |
| 2. Need help feeding yourself | No=0, Yes=1 |
| 3. Need help dressing yourself | No=0, Yes=1 |
| 4. Need help using the toilet | No=0, Yes=1 |
| 5. Need help with housekeeping | No=0, Yes=1 |
| 6. Need help climbing stairs | No=0, Yes=1 |
| 7. Need help bathing | No=0, Yes=1 |
| 8. Need help walking | No=0, Yes=1 |
| 9. Need help using transportation | No=0, Yes=1 |
| 10. Need help getting in and out of bed | No=0, Yes=1 |
| 11. Need help managing medications | No=0, Yes=1 |
| 12. Depend on assistive devices (walker, cane, etc) or other people to perform activities of daily life | No=0, Yes=1 |
| 13. Dependent on device for normal breathing | No=0, Yes=1 |
| 14. Climb 2 flights of stairs without rest | No, can`t at all=1  Yes, with difficulty=0.5  Yes, with no difficulty=0 |
| 15. Myocardial infarction | No=0, Yes=1 |
| 16. Diabetes | No=0, Yes=1 |
| 17. Peripheral vascular disease | No=0, Yes=1 |
| 18. Cerebrovascular disease | No=0, Yes=1 |
| 19. Dementia | No=0, Yes=1 |
| 20. Chronic obstructive pulmonary disease | No=0, Yes=1 |
| 21. Peptic ulcer | No=0, Yes=1 |
| 22. Hemiplegia/paraplegia | No=0, Yes=1 |
| 23. Renal disease | No=0, Yes=1 |
| 24. Moderate/severe liver disease | No=0, Yes=1 |
| 25. Rheumatologic disease | No=0, Yes=1 |
| 26. Hypertension | No=0, Yes=1 |
| 27. Hyperlipidemia | No=0, Yes=1 |
| 28. Body mass index | Underweight/obese=1  Overweight=0.5  Normal=0 |
| 29. Depression | No=0, Yes=1 |
| 30. Anemia | No=0, Yes=1 |
| 31. Metastatic solid tumor | No=0, Yes=1 |
